# Do cats receiving pre-appointment trazodone experience less stress during veterinary visits?

**DOI:** 10.18849/ve.v10i1.694

**Published:** 2025-02-17

**Authors:** Stephanie Shone

**Keywords:** aggression, anxiety, behaviour, cat, compliance, fear, feline, stress, trazodone

## Abstract

**PICO question:**

In cats does pre-appointment trazodone administration, compared to no trazodone, effectively reduce patients’ stress during veterinary visits?

**Clinical bottom line:**

**Category of research:**

Treatment.

**Number and type of study designs reviewed:**

Two studies were identified and reviewed. One was a double blind, placebo controlled, randomised crossover study. The other was a blinded placebo controlled randomised crossover study.

**Strength of evidence:**

Weak.

**Outcomes reported:**

In the first appraised paper 10 cats with ≥ 1 behavioural sign consistent with transport or veterinary examination associated anxiety were randomly assigned and orally administered a 50 mg trazodone tablet or a placebo (a sodium bicarbonate tablet identical in appearance to a trazodone tablet).

9/10 cats had anxiety-related signs during the veterinary visit when given the placebo, whereas 3/10 had them when given trazodone. Overall, behavioural scores assigned by the veterinarian were significantly (Wilcoxon signed rank test, P = 0.006) lower for cats after receiving trazodone versus the placebo. Trazodone administration resulted in significantly (Wilcoxon signed rank test, P = 0.01) more favourable tractability scores than with the placebo administration, with cats considered relaxed versus tense after receiving trazodone. For all components of the veterinary examination, trazodone administration resulted in significantly (McNemar test, P = 0.03 for each comparison) lower stress scores before, during and after veterinary examination. However, one cat was reported to become more fearful and vocal after trazodone administration.

The second paper assessed the sedative and anxiolytic effects of 50 mg, 75 mg, and 100 mg trazodone administration to 6 purpose-bred, male, neutered laboratory cats, prior to and during a veterinary examination to a placebo (food or treats with no tablet). Since the 50 mg and 75 mg doses were not randomised the author of this Knowledge Summary did not include them as part of the statistical analysis. This study found no statistically significant difference in scoring between the effects of the 100 mg trazodone and the placebo, after analysis of the total behavioural and mean overall stress scores. There was a statistically significant difference in enclosure activity with lower levels of activity in cats administered 100 mg trazodone compared to the placebo. However, this is likely more reflective of the sedative effects of trazodone rather than anxiolysis.

**Conclusion:**

The first appraised paper found promising evidence that trazodone could be effective at reducing stress in cats during veterinary visits. However, despite the strong study design, many limitations were identified, most notably the small sample size and large number of uncontrolled variables. This undermined the statistically significant data produced. The second study did not produce statistically significant evidence that trazodone ameliorates stress during a veterinary visit.

A conclusion to the PICO question cannot be made due to lack of relevant studies and statistically significant data. Evidence found supporting the PICO question is weak and therefore more studies with larger sample sizes and less variability are needed to determine the efficacy of trazodone use to reduce stress in cats during veterinary visits.

## How to apply this evidence in practice

The application of evidence into practice should take into account multiple factors, not limited to: individual clinical expertise, patient’s circumstances and owners’ values, country, location or clinic where you work, the individual case in front of you, the availability of therapies and resources.

Knowledge Summaries are a resource to help reinforce or inform decision-making. They do not override the responsibility or judgement of the practitioner to do what is best for the animal in their care.

## The Evidence

Stevens et al. (2016) produced promising evidence that 50 mg trazodone administration pre-appointment reduces stress in anxious cats during veterinary visits. This conclusion is based on the good study design and statistically significant results produced. The study was double blind and placebo controlled which reduced bias by blinding both the veterinarian and technician and the owner to whether trazodone or the placebo had been administered to the animal being scored. In addition, the crossover design meant that each animal received and was scored after both the trazodone and placebo were given following a washout period of 1 to 3 weeks in-between each treatment. This reduced biological variation. This type of study is classified near the top of the evidence hierarchy. However, a notable limiting factor to this study was the small sample size of only 10 reducing the reliability of the data collected.

Orlando et al. (2016) had a similar study design to Stevens et al. (2016). In this study the observer was blinded to the treatment administered. The 100 mg trazodone and placebo were randomly assigned, and the effects compared. The sample size was also limited with only 6 cats reducing the reliability of the results. This study did not produce statically significant data that supports the use of trazodone for anxiolysis in cats.

Overall, the evidence is weak and further studies with a larger sample size based on sample calculation and fewer variables are needed to obtain more representative and statistically significant results before an evidence-based conclusion can be made. Furthermore, a dosing regimen needs to be established with prevalence of adverse reactions considered to establish whether the benefit of anxiolysis is outweighed.

## Summary of the Evidence

**Orlando et al. (2016):** Use of oral trazodone for sedation in cats: a pilot study **Aim: **to assess the efficacy and safety of a single dose of oral trazodone on sedation in cats.

Population	Healthy client owned exclusively indoor cats between 2–12 years of age with a history of anxiety during transport or veterinary examination. Cats were required to have ≥ 1 behavioural sign consistent with transport- or veterinary examination-associated anxiety. Three castrated males and seven spayed females qualified for and completed the study with a mean age of 6.8 years (range 2.1 to 10.7 years) and a mean body weight of 4.6 kg (range 3.3 to 6.5 kg). All cats were reported by their owners to have had at least one of the evaluated signs of anxiety during transport in a carrier in the past. Eight cats were reported by their owners to have had at least one of the evaluated signs of anxiety during veterinary examination in the past.
Sample Size	Of 13 cats, 10 completed the trial—two cats were withdrawn due to illness unrelated to the study protocol and another was withdrawn by the owner after reporting a suspected adverse effect to the trazodone administration.
Intervention Details	Each cat was assigned to first receive a single dose of 50 mg trazodone hydrochloride or a placebo (a sodium bicarbonate tablet identical in appearance to trazodone) by mouth. A randomisation table was used to establish which cat received which treatment first and after a 1–3-week washout period each cat received the opposite treatment. Owners were instructed to orally administer the randomly assigned treatment (placebo or 50mg trazodone hydrochloride) in a manner that would be best tolerated by their cat as decided by each cat’s respective owner. Treatment was provided to 4 cats in malleable treats, to 4 cats hidden in canned food, and to 2 cats by direct administration by hand. All owners reported that their cats received the total amount of the medication. Trazodone doses ranged from 7.7–15.2 mg/kg. The duration between treatment administration at home and placement of the cat into the travel carrier was 1–1.5 hours. Owners lived within a 10–30-minute drive of the veterinary After a brief wait in the lobby (~1.5–2 hours post treatment administration) the cat and owner were escorted to a designated examination room. The cat carrier was placed on the floor and the door opened to allow the cat to voluntarily exit. If the cat exited voluntarily, it was then allowed to explore the room briefly then placed on the examination table. If the cat did not exit voluntarily the carrier was placed on the examination table, the top removed, and the cat was gently lifted onto the table. If the carrier top could not be removed the cat was gently extracted from the carrier and placed on the examination table. An identical physical examination was performed on each cat by the same veterinarian and assisted by the same technician in the owner’s presence throughout the study. The owners, veterinarian, and technician were all blinded to which treatment had been administered to each cat. The physical examination consisted of 12 procedures typically completed within 20 minutes and in the same order. This involved removal of the cat from the carrier; placement on the examination table; weighing on a tabletop scale; visual examination of the head, eyes, and oral cavity without instrumentation; thoracic auscultation and measurement of heart and respiratory rates; abdominal palpation; lymph node palpation; measurement of aural temperature; direct ophthalmoscopic examination; otoscopic examination; shaving of a small section of hair at base of tail and Doppler ultrasonographic determination of arterial blood pressure; and release after examination. The physical examination concluded ~2–2.5 hours post treatment administration.
Study design	Double blind, placebo-controlled, randomised crossover study.
Outcome studied	3 scoring systems were used to assess for signs of anxiety and fear at specific points: the McCune cat stress score (CSS), the tractability score, and the behavioural response score. Owners used a standard form to assign cumulative stress scores to their cat at the following points: before transport, during transport, and after transport, while in the clinic waiting room, during the examination, and immediately after the examination. Objective assessments: Owners were asked to report for each assessment point the presence or absence of objective signs associated with anxiety, including urination, defaecation, anal gland release, vomiting, excessive salivation, trembling, open-mouth breathing, and vocalisation. Physiological data was collected by the veterinarian during the examination including heart rate, respiratory rate, aural temperature, and Doppler ultrasonographic measurement of arterial blood pressure. Subjective assessments: Intensity of vocalisation was scored (0 = absent, 1 = mild, 2 = moderate, and 3 = severe) by the owner at each assessment point. Behavioural data following each of the 12 procedures and for the examination overall were assigned by the veterinarian by use of the behavioural response score system^.^ (0 = absent, 1 = mild, 2 = moderate, 3 = severe, and 4 = too severe to complete), which involved scoring 4 specific cat behaviours at each assessment point: vocalisation (whimper, cry, or meow), struggling or attempts to escape, aggression (except for hissing or growling; ears flattened back, tail lash, or batting), and severe aggression (biting, attacking, and hissing and growling). The veterinarian assigned a cumulative stress score for the entire examination (1 = fully relaxed, 2 = weakly relaxed, 3 = weakly tense, 4 = very tense, 5 = fearful or stiff, 6 = very fearful, and 7 = terrorised). At the conclusion of the veterinary examination, both the veterinarian and owner independently assigned a tractability score, which was based on the ease by which the veterinarian could perform each examination procedure and the appearance of the cat (0 = cat completely relaxed, 1 = easy to examine but not entirely relaxed, 2 = relatively easy to examine, 3 = restraint needed to examine safely, 4 = very difficult to examine, and 5 = unable to examine). At the conclusion of each visit and to understand owner perception of their cat's anxious behaviour, owners were asked to speculate whether their cat received the placebo or trazodone. After each visit, in a survey sent via email, owners were asked to report any adverse events noted within 24 hours after administration of the assigned treatment (placebo or 50mg trazodone hydrochloride).
Main findings (relevant to PICO question)	Owner assessment: After receiving either treatment no cats had any of the evaluated signs of anxiety. Stress scores assigned before transport (after treatment administration 1 to 1.5 hours earlier) were significantly lower for trazodone than for the placebo (McNemar test, P = 0.02) During transport, 3 cats had 1 or more of the evaluated signs of anxiety. One cat urinated and defaecated after receiving the placebo and urinated after receiving trazodone. The second cat vomited, had hypersalivation, trembled, and breathed with an open mouth after receiving the placebo. The third had open mouth breathing after receiving the placebo. Stress scores assigned during transport did not differ significantly between treatments. After transport while in the waiting room, no cats had any evaluated sign of anxiety after receiving trazodone; however, 1 cat urinated, defaecated, excessively salivated, and had open mouth breathing and another cat excessively salivated after receiving the placebo. Trazodone administration resulted in significantly (McNemar test, P = 0.02) lower cat stress scores at this assessment point than did placebo administration. 10 cats vocalised before transport when given the placebo, and 7 cats vocalised when given trazodone; all except 1 cat had a reduction in their vocalisation intensity score. Trazodone administration resulted in significantly (Wilcoxon signed rank test, P = 0.008) less frequent vocalisation during transport than did placebo administration. 8 cats vocalised after transport when given the placebo, and 2 vocalised when given trazodone. During the veterinary examination, no cats urinated, defaecated, released their anal glands, vomited, excessively salivated, trembled, or had open-mouth breathing, regardless of treatment received. No cats vocalised. Trazodone administration resulted in significantly (Wilcoxon signed rank test, P = 0.04) lower stress scores during this period than did placebo administration. Owner-assigned tractability scores during the examination were significantly (P = 0.03) more favourable with trazodone versus the placebo. After the veterinary examination, no cats urinated, defaecated, released their anal glands, vomited, excessively salivated, trembled, or had open-mouth breathing, regardless of treatment received. Trazodone administration resulted in significantly (Wilcoxon signed rank test, P = 0.008) lower stress scores during this period than did placebo administration. Although blinded to treatment, 9 of 10 owners correctly identified the treatment their cats received after observing their behaviour during transport and examination. Veterinary assessment: All physiologic values for all cats were within reference ranges after trazodone and placebo administration. No significant differences were identified between trazodone and placebo with respect to heart rate (P = 0.55), blood pressure (P = 0.91), or aural temperature (P = 0.24). Respiratory rate was lower, albeit nonsignificantly (P = 0.055), after trazodone versus placebo administration. Overall, behavioural scores as assigned by the veterinarian were significantly (Wilcoxon signed rank test, P = 0.006) lower for cats after receiving trazodone versus the placebo. Trazodone administration resulted in significantly (Wilcoxon signed rank test, P = 0.01) more favourable tractability scores than did placebo administration, with cats considered relaxed versus tense after receiving trazodone. For all components of the veterinary examination, trazodone administration resulted in significantly (McNemar test, P = 0.03 for each comparison) lower stress scores before, during, and after veterinary examination. Adverse events: In response to the email survey after the veterinary visit, owner reports of adverse events were limited to 1 report of transient sleepiness (in addition to the one cat that was withdrawn by the owner from the study due to agitation and vocalisation 30 minutes post trazodone administration). One cat was scored as fearful when given the placebo and very fearful (a worse score) when given trazodone. Because anxious cats without treatment may be fearful or very fearful for veterinary visits, this particular cat's behaviour may have represented a lack of response to trazodone or a paradoxical response.
Limitations	There is no calculated sample size. The use of sample size calculation directly influences research findings. Very small samples undermine the internal and external validity of a study. Very large samples tend to transform small differences into statistically significant differences—even when they are clinically insignificant. As a result, both researchers and clinicians are misguided, which may lead to failure in treatment decisions. Small sample size: only 10 cats completed the study. A small sample size reduces the significance and undermines the validity of the study because the results are less representative of the entire population and there is a higher margin of error. Large variability in sample population in terms of breed, age, entire vs neutered, home environment, and sex. This is due to the absence of clear inclusion and exclusion criteria leading to a broad range of eligible cats. Variability in method of treatment administration resulting in different levels of stress imposed on each cat in treats, in canned food, and by manual administration. In addition, the presence of food in the upper gastrointestinal tract in some cats and not others lead to variability of the time taken for absorption of the trazodone into the blood. Variability in the duration of time from administration of treatment to placing the cat in the carrier (1 to 1.5 hours) and variability in the length of time the cat is transported (10 to 30 minutes). This results in variability of time from administration of treatment to time of the physical examination being carried out by the veterinarian (1.5 to 2 hours) and therefore absorption levels at each assessment point will have varied between cats. Lack of journey detail included—quiet vs busy roads, frequent starting and stopping, the position of the carrier in the car (back seat, footwell, in the boot etc.). Variability in these factors could impact levels of stress endured by the cat while being transported. Variability in doses used per kg trazodone doses ranged from 7.7 to 15.2 mg/kg. Variability in how cats exited the carrier; some exited on their own, others were removed via removing the top of the carrier and others were extracted from the carrier via the door resulting in different levels of stress imposed on the cat depending on which method was used. Variability in the washout period was between 1 to 3 weeks. Mostly subjective scoring was used, especially by the owners. Objective scoring by owners did not involve measurement of physiological signs of anxiety such as heart rate or respiratory rate. These were only assessed by the veterinarian in the clinic and so there is no data for these parameters at other assessment points. Owners may have given higher scores on the subjective assessments if they were stressed themselves. None of the scoring systems used are validated. The McCune cat stress score has been regarded as a static and subjective measurement of behaviours displayed over the short term. It has extensively been used to evaluate the level of welfare for cats in shelters. However, it has been found that the CSS might not clearly correlate with physiological stress, as seen by difficulties to validate the scores, but it may be an indication of coping with environmental challenges in cats. The study does not explore the effects of other doses of trazodone, only 50 mg. Lack of reported detail within the veterinary clinic such as how many other people/animals there are in the lobby (i.e. how busy), how loud it is, what species of animals are present, the size of the other animals etc. as this could contribute to stress levels experienced by the cat. Type of carrier is not specified. Details such as size of carrier compared to cat and whether a blanket was used to protect from visual stimuli. Information on where the carrier was placed is not included, i.e. on the floor with potential olfactory stressors or on the owner’s lap, on a table etc.

Table note...

**Stevens et al. (2016):** Efficacy of a single dose of trazodone hydrochloride given to cats prior to veterinary visits to reduce signs of transport- and examination-related anxiety **Aim: **to determine the effect of a single dose of trazodone hydrochloride on reducing anxiety levels during transport and a veterinary examination in cats.

Population	Purpose bred, neutered male, domestic shorthair laboratory cats aged 6 months (1 cat) to 3–5 years old (5 cats). Cat weights ranged from 3 kg to 4.7 kg. The five adult cats had previously been used as control subjects for a separate study where they had been conditioned to entering a carrier voluntarily. The 6-month-old cat had not received any prior conditioning.
Sample Size	6 cats.
Intervention Details	Each cat was kept in identical wire enclosures of the same dimensions containing the same items in the same room within a laboratory facility and were kept on a 12/12 hour light/dark cycle. Each cat was fed Hill’s™ Science Diet™ Adult Light two times daily and maintained at an ideal body condition score (5/9) based on the Purina® Body Condition System. Prior to enrollment each cat received a physical examination, chemistry panel, complete blood count, and urinalysis. After the study, these tests were repeated to rule out any changes attributable to administration of trazodone. Cats were administered treatments over a 4-week period. A 50 mg trazodone tablet (range: 10.6–16.7 mg/kg) was given as the first treatment and 75 mg trazodone tablet (range: 16.0–25 mg/kg) as the second treatment. These treatments were not randomised and so the data obtained has not been statistically analysed or evaluated as part of this summary. The third and fourth treatments were randomised to either the 100 mg trazodone tablet (range: 21.3–33.3 mg/kg doses) or placebo (treats or food with no tablet). The washout period between treatment days was 4–7 days. Trazodone tablets were quartered and hidden in a tablespoon of Hill’s™ Prescription Diet a/d or Pill Pockets™. On days when treatment was administered the cats were fed a reduced portion (1/8 cup) approximately 3 hours before treatment. Examinations consisted of the following and were carried out 90 minutes after treatment administration: A carrier was placed outside the enclosure door, which was opened, and the cat was allowed to enter voluntarily, with the incentive of a treat or if refusing was gently picked up and placed in the carrier. The cat was then carried to the examination room (35 m down the hall) and the carrier was placed on an examination table. The cats were allowed to exit voluntarily or removed from the carrier via removal of the top. If resistant to removal they were left in place. Each physical examination was performed in the same order: cardiac and pulmonary auscultation followed by peripheral lymph node palpation, abdominal palpation, otoscopic examination, ophthalmoscopic examination, oral examination, mock jugular venepuncture in a sternal position and mock cystocentesis performed in lateral recumbency. All cats were handled with minimal restraint using a towel or technician assistance if necessary. Treats were given to all cats to encourage compliance. If a cat became too fractious, the examination was concluded. Each cat was given the chance to enter the carrier voluntarily before being returned to their enclosure.
Study design	Blinded placebo controlled randomised crossover study.
Outcome studied	Objective assessments: Accelerometer activity monitors: Each cat was fitted with a pre-equilibrated activity monitor attached to its collar to record locomotor activity. The monitor was applied 1 week prior to the study to allow a period of acclimation to the device. The monitor was worn, and data was collected for the 4-week duration of the study and monitors were set to record at intervals of 30 seconds. Physiological parameters: heart rate and respiratory rate were recorded during the veterinary examination. Subjective assessments: Activity observations: Focal observation samples of the cats’ activity in their enclosures were recorded by a handheld digital video camera prior to treatment administration and every 30 minutes for 4 hours after. Each cat was given an enclosure activity score from 1 (sedate) to 5 (very active) for each time interval. Behavioural response to examination: This was performed for each cat for each treatment. All examinations and behavioural ratings were performed by an investigator masked to treatment. No information can be found on the validity of this scoring system. McCune cat stress score: Cats were assessed at three time points: while in their carriers prior to the examination (pre-examination), out of the carrier during the examination (examination), and in their carrier after examination (post-examination). These scores were combined for an overall stress score. This was performed for each cat for each treatment.
Main findings (relevant to PICO question)	Accelerometer data revealed that cats were less active after receiving 100 mg trazodone tablet compared to the placebo with peak sedation occurring 2.5 hours post administration. Enclosure activity scores of 1 (‘sedate’) and 2 (‘calm’) were only observed in cats when they received trazodone (at any of the doses) with 4/6 of the cats having a more than two-point reduction in their scores after being given a 100 mg dose. There was a statistically significant difference between the enclosure activity scores of cats when they received trazodone 100 mg compared with placebo (Fisher’s exact test, n = 12; P = 0.06). Total behavioural response scores given for each cat reflected only struggling, aggression and vocalisation. The scores revealed no significant difference between cats that received trazodone 100 mg and placebo (Wilcoxon signed rank test, n = 6; P = 0.594). There was no difference between the 100 mg trazodone and placebo groups for the overall stress scores recorded before, during and after the examinations (Wilcoxon signed rank test, n = 6; P = 1.000).
Limitations	There is no calculated sample size. The use of sample size calculation directly influences research findings. Very small samples undermine the internal and external validity of a study. Very large samples tend to transform small differences into statistically significant differences—even when they are clinically insignificant. As a result, both researchers and clinicians are misguided, which may lead to failure in treatment decisions. Small sample size: only 6 cats were involved in the study. A small sample size reduces the significance and undermines the validity of the study because the results are less representative of the entire population and there is a higher margin of error. The McCune cat stress score is not a validated scoring system, but it has been regarded as a static and subjective measurement of behaviours displayed over the short term (Broadley et al., 2014). It has extensively been used to evaluate the level of welfare for cats in shelters. The 50 mg and 75 mg doses were not randomised. The washout period ranged from 4–7 days. Subjects were limited to neutered male cats. Cats were house in a single room with visual access to the other cats. It is possible that cats were influenced by the stimuli of the other cats’ activities One cat did not receive a full dose of the 100 mg trazodone on one treatment day. Not all cats received the medication in similar types or amounts of food and some were given additional treats as an incentive which will have altered absorption rates. Not all cats entered and exited the carrier voluntarily resulting in different stress levels imposed on each cat. It is difficult to apply the finding from this study in clinical practice as the cats are all purpose bred laboratory cats and so likely to behave differently to non-laboratory cats. In addition, it has not been stated that any of the cats had pre-existing signs of veterinary visit related anxiety.

Table note...

## Appraisal, application and reflection

Cats commonly show signs of anxiety during veterinary visits, increasing the possibility of aggressive behaviours and/or resistance during examination. This in turn not only has a negative mental and potentially physical impact on the cat itself, but also the owners and veterinary staff involved (Rodan, 2010). Owners’ perception of stress in their cats is a primary reason for failing to seek veterinary care. This can be detrimental to the health and welfare of the cat (van Haaften et al., 2017). Low stress environments and handling alone may not significantly lower anxiety, but the addition of behavioural medications may aid in mitigating anxiety and fear associated with veterinary care and therefore improve owner compliance and animal welfare (Erickson et al., 2021).

Trazodone is a second generation triazolopyridine derivative that can be used off license for the management of anxiety in cats. It is classified as a serotonin receptors antagonist and reuptake inhibitor (SARI) (Gruen & Sherman, 2008) owing to its primary pharmacological mechanism as an antagonist at serotonin 2A receptors and its secondary mechanism as a serotonin reuptake inhibition (Gilbert-Gregory et al., 2016). In comparison, selective serotonin reuptake inhibitors (SSRI), another commonly used classification of anti-depressant, inhibit reuptake of serotonin through inhibition of the serotonin transporter but they do not antagonise the serotonin receptors. Trazodone is also a potent alpha-adrenoreceptor and histaminergic antagonist and possesses anxiolytic and hypnotic properties. It has been used as an anti-depressant in humans since the 1970s and has also been found to be beneficial for many other medical and psychiatric conditions including anxiety (Chea & Giorgi, 2017). In dogs it has been used to promote low stress handling during a veterinary visit, as well as to treat behavioural disorders and facilitate postsurgical confinement (Gilbert-Gregory et al., 2016).

Stevens et al. (2016) compared the anxiety levels of 10 cats with known veterinary visit related anxiety and the effect of a single 50 mg tablet of trazodone compared to a placebo administered by the owner prior to the visit. No confidence intervals were reported so values of P < 0.05 have been considered significant. Owner assigned stress scores during transport did not differ significantly, however, it was found that trazodone administration resulted in significantly (Wilcoxon signed rank test, P = 0.008) less vocalisation. Owner stress scores were significantly lower after trazodone administration compared to the placebo at the majority of points; before transport/1 to 1.5 hours post administration (McNemar test, P = 0.02), after transport/in the waiting room (McNemar test, P = 0.02), during the veterinary examination (Wilcoxon signed rank test, P= 0.04) and after the vet examination (Wilcoxon signed rank test, P = 0.008). In addition, owner assigned tractability scores during the examination were significantly (P = 0.03) more favourable with trazodone versus placebo. It is also important to note that although the owners were blinded to which treatment their cat was receiving, 9 out of 10 correctly identified the treatment after observing their cat’s behaviour and several requested subsequent trazodone for their cat for future veterinary visits. Overall, these results provide promising evidence that trazodone does help to reduce stress in anxious cats during veterinary visits.

This study (Stevens et al., 2016) has a strong study design for multiple reasons. The comparison of a placebo to the trazodone treatment reduced biological variation. In addition, the fact that the treatments were randomised, and the owners, veterinary staff were all blinded to the administered treatment eliminated any potential bias. The strength of the study design is, however, undermined by its limitations. Firstly, the small sample size (n = 10) means the results are less likely to be representative. Moreover, there was a broad range of eligible cats due to absence of a clear inclusion and exclusion criteria leading to a lot of variability in the studied population. Three eligible cats were excluded from the study. One of these cats one was believed to have had an adverse event, becoming more vocal and agitated after the trazodone administration, and was subsequently withdrawn by the owner. The other two cats were withdrawn prior to the study commencing. An email survey was sent to owners asking them to report any adverse effects within 24 hours after administration of the treatment. There was only one report of transient sleepiness. No vomiting, diarrhoea or behavioural changes were reported. A larger sample size would also be useful to establish the incidence of side effects and therefore the safety index of trazodone at the dosage used and others.

Assessment points in this study (Stevens et al., 2016) were dictated by certain activities rather than time since trazodone administration. Therefore, the time from administration of treatment to the cat being put in the carrier, travelling in the car, and being assessed by the veterinarian varied. This will have resulted in different absorption levels of trazodone in the blood of each cat at each assessment point. In addition, absorption levels will have varied based on how much food the cat had in its stomach and the dose it received relative to its weight as a 50 mg dose was given to all cats regardless of weight. Despite the cats all receiving the same physical examination the cats would have been subject to different levels of potential stressors at multiple points throughout the study. For example, variations in the length and condition of the journey to the veterinary clinic (lots of starting and stopping, windy roads vs a short journey with few turns and stops, position in car the carrier was placed during transport), stressful stimuli in the waiting room both auditory, visually and olfactory, type of carrier used, time in the waiting room and method of entering and exiting the carrier in the examination room (voluntary vs extraction via the door or roof). This study used both subjective and objective scoring systems. None of the scoring systems have been validated. The subjective scores, especially by the owners, will have been influenced by person feelings and therefore it is impossible to ensure consistency. This will also have been influenced by the level of stress experienced by the owner. It would have also been helpful for more objective scoring of physiological parameters such as respiration rate to be done by owners throughout the study. Another important inconsistency is the washout period used between treatments which varied between 1–3 weeks.

Orlando et al. (2016) investigated the sedative and behavioural effects of 50 mg, 75 mg, 100 mg trazodone, and a placebo on purpose bred laboratory cats during a veterinary examination. Because the 50 mg and 75 mg doses of trazodone were not randomised, the data obtained during these treatment days was excluded from statistical analysis and not taken into consideration in this evidence summary. Data from trazodone 100 mg and placebo treatments were compared and because of the small sample size, a p-value < 0.1 was considered significant. Heart rates (Wilcoxon signed rank test, n = 5; P = 0.5) and respiratory rates were not significantly affected by trazodone 100 mg compared with placebo (Wilcoxon signed rank test, n = 4; P = 0.375). Enclosure activity scoring analysis revealed that there was a statistically significant difference between the enclosure activity scores of cats when they received trazodone 100 mg compared with placebo (Fisher’s exact test, n = 12; P = 0.06). However, this likely reflects the sedative effects of trazodone rather than its anxiolytic effect. Total behavioural response scores revealed no significant difference between cats that received trazodone 100 mg and placebo (Wilcoxon signed rank test, n = 6; P = 0.594). The mean overall stress score for before, during and after examination was the same between the trazodone 100 mg and placebo groups (Wilcoxon signed rank test, n = 6; P = 1.000). Overall, this study does not provide evidence that the administration to cats before a veterinary visit would be beneficial to reduce anxiety.

Both studies (Stevens et al., 2016 and Orlando et al., 2016) used a placebo control and the McCune cat stress scoring systems and behavioural response scoring system in addition to assessment of physiological parameters. They both also scored the cats before, during, and after a veterinary examination. Because laboratory cats were used in this study (Orlando et al., 2016) it was possible to reduce variability significantly. The cats were all in the same environment being fed the same diet and the scoring was carried out at the same time interval post treatment administration per cat. The observer was blinded to the treatment that had been administered therefore reducing bias. Also, the cats were transported in the same carriers and underwent the same journey from their kennel to the examination room. This helped ensure that no external factors varied the amount of stress each animal underwent. There was however variation in how the cats exited and entered the carriers and the author does state that there was visual access between cats, which could have influenced their activities. Other limitations stated by the author were that not all the cats received the medication in similar types of food which may have had an impact on absorption rates. In addition, one cat did not receive a full dose on one treatment day (100 mg) and administration of the 50 mg and 75 mg doses were not randomised. There was also variability in the washout period; between 4 to 7 days. A large limitation was that 3 out of 5 of the outcomes measured were subjective and not validated. Both objective outcomes assessed could have been decreased as a result of sedation rather than anxiolysis. Despite the benefits of using laboratory cats to reduce the number of variables in the study, including biological variation, it also meant that the sample population was not representative of a ‘normal’ domesticated cat and so is less applicable in practice. The small sample size also means that the results are less likely to be significant and representative. In terms of safety no abnormalities were detected on physical examination and no cats had any adverse effects. Laboratory values were not appreciably different from values obtained prior to the onset of the study.

## Methodology

**Search Strategy:** 

Databases searched and dates covered	CAB Abstracts on the OVID interface 1973 to 2 April 2024 PubMed accessed via the NCBI website 1920 to 2 April 2024
Search terms	CAB Abstracts: cat or cats or feline* or queen* or tom or toms trazodone or desyrel or molipaxin or oleptro anxiety or anxious or fear or fearful or stress or stressed or stressful or distressed or distress or panic or phobi* or nervous or nervousness or vocalise* or urinat* or hide or hides 1 and 2 and 3 PubMed: cat or cats or feline or queen or tom or tom trazodone or desyrel or molipaxin or oleptro anxiety or anxious or fear or fearful or stress or stressed or stressful or distressed or distress or panic or phobia or nervous or nervousness or vocalise or urinate or hide or hides 1 and 2 and 3
Dates searches performed:	02 April 2024

**Exclusion / Inclusion Criteria:** Caption placeholder

Exclusion	Papers inappropriate to the PICO: non-related title or abstract. Any other species but cats. No direct comparison between use of trazodone and without trazodone/placebo.
Inclusion	Effects investigated in a veterinary setting. Papers in English. Cats aged 6 months or older. Cats weighing more than 1kg.

Table note placeholder

**Search Outcome:** Caption placeholder

Database	Number of results	Excluded – inappropriate to PICO	Excluded –wrong species	Total relevant papers
CAB Abstracts	7	4	1	2
PubMed	10	8	0	2
Total relevant papers when duplicates removed				2

Table note placeholder

## ORCID

Stephanie Shone: https://orcid.org/0009-0004-9025-8474

### Conflict of Interest

The author declares no conflicts of interest.

## Disclaimer

Knowledge Summaries are a peer-reviewed article type which aims to answer a clinical question based on the best available current evidence. It does not override the responsibility of the practitioner. Informed decisions should be made by considering such factors as individual clinical expertise and judgement along with patient’s circumstances and owners’ values. Knowledge Summaries are a resource to help inform and any opinions expressed within the Knowledge Summaries are the author’s own and do not necessarily reflect the view of the RCVS Knowledge. Authors are responsible for the accuracy of the content. While the Editor and Publisher believe that all content herein are in accord with current recommendations and practice at the time of publication, they accept no legal responsibility for any errors or omissions, and make no warranty, express or implied, with respect to material contained within. For further information please refer to our Terms of Use.

## References

[BIBR-1] (2014). Effect of single-cat versus multi-cat home history on perceived behavioral stress in domestic cats (Felis silvestrus catus) in an animal shelter. Journal of Feline Medicine and Surgery.

[BIBR-2] Chea B., Giorgi M. (2017). Trazodone: A Review of Its Pharmacological Properties and Its Off-Label Use in Dogs and Cats. American Journal of Animal and Veterinary Sciences.

[BIBR-3] Erickson A., Harbin K., MacPherson J., Rundle K., Overall K.L. (2021). A review of pre-appointment medications to reduce fear and anxiety in dogs and cats at veterinary visits. The Canadian Veterinary Journal.

[BIBR-4] Faber J., Fonseca L. (2014). How sample size influences research outcomes. Dental Press Journal of Orthodontics.

[BIBR-5] Gilbert-Gregory S.E., Stull J.W., Rice M.R., Herron M.E. (2016). Effects of trazodone on behavioral signs of stress in hospitalized dogs. Journal of the American Veterinary Medical Association.

[BIBR-6] Gruen M.E., Sherman B.L. (2008). Use of trazodone as an adjunctive agent in the treatment of canine anxiety disorders: 56 cases (1995–2007. Journal of the American Veterinary Medical Association.

[BIBR-7] Gruen M.E., Thomson A.E., Clary G.P., Hamilton A.K., Hudson L.C., Meeker R.B., Sherman B.L. (2013). Conditioning laboratory cats to handling and transport. Lab Animal.

[BIBR-8] Jaeger G.H., Marcellin-Little D.J., DePuy V., Lascelles B.D.X. (2007). Validity of goniometric joint measurements in cats. American Journal of Veterinary Research.

[BIBR-9] Kessler M.R., Turner D.C. (1997). Stress and Adaptation of Cats (Felis Silvestris Catus. Housed Singly, in Pairs and in Groups in Boarding Catteries. Animal Welfare.

[BIBR-10] McCune S. (1992). Temperament and the welfare of caged cats.

[BIBR-11] Orlando J.M., Case B.C., Thomson A.E., Griffith E., Sherman B.L. (2016). Use of oral trazodone for sedation in cats: a pilot study. Journal of Feline Medicine and Surgery.

[BIBR-12] Purina Body Condition System. https://www.purinainstitute.com/centresquare/nutritional-and-clinical-assessment/purina-body-condition-system.

[BIBR-13] Rand J.S., Kinnaird E., Baglioni A., Blackshaw J., Priest J. (2002). Acute Stress Hyperglycemia in Cats is Associated with Struggling and Increased Concentrations of Lactate and Norepinephrine. Journal ofVeterinary Internal Medicine.

[BIBR-14] Rodan I. (2010). Understanding Feline Behavior and Application for Appropriate Handling and Management. Topics in Companion Animal Medicine.

[BIBR-15] Stevens B.J., Frantz E.M., Orlando J.M., Griffith E., Harden L.B., Gruen M.E., Sherman B.L. (2016). Efficacy of a single dose of trazodone hydrochloride given to cats prior to veterinary visits to reduce signs of transport- and examination-related anxiety. Journal of the American Veterinary Medical Association.

[BIBR-16] Haaften K.A., Forsythe L.R.E., Stelow E.A., Bain M.J. (2017). Effects of a single preappointment dose of gabapentin on signs of stress in cats during transportation and veterinary examination. Journal of the American Veterinary Medical Association.

